# High-dose chemotherapy of metastatic breast cancer: the end of the beginning?

**DOI:** 10.1038/bjc.1997.81

**Published:** 1997

**Authors:** J. Crown


British Journal of Cancer (1997) 75(4), 467–469
© 1997 Cancer Research Campaign

Editorial

### High-dose chemotherapy of metastatic breast cancer: the end of the beginning?

**J Crown**

St. Vincent's Hospital, Dublin 4, Republic of Ireland

**Keywords:** high-dose chemotherapy; breast cancer

Despite a diverse armamentarium of active drugs and response rates that now exceed 70%, the survival impact of chemotherapy in metastatic breast cancer is limited, and the disease remains essentially incurable (Cold et al, 1992). This frustrating, partial chemosensitivity prompted investigators to explore potential clinical applications of the relationship that had been demonstrated in laboratory models between drug dose and anti-cancer effect (Teicher et al, 1988). Random assignment trials, in which variations in dose within the 'standard' range (i.e. doses that could be administered without specialized haematopoietic support) were studied, yielded modest and inconsistent results; however the degree of dose escalation that had been attempted in these trials was relatively minor in comparison with that achieved in the laboratory (Hortobagyi et al, 1987; Bastholt et al, 1996).

The development of marrow autografting facilitated the study of very high doses of some drugs, prominently the alkylating agents, and allowed clinical investigators to mimic the levels of drug exposure that had been assayed in the preclinical systems. The first trials of high-dose chemotherapy with autograft support in breast cancer were performed in patients with disease that had failed conventional treatment. While high rates of response were achieved, indicating that dose escalation could indeed partly overcome drug resistance, these responses were of brief duration. In addition treatment-related mortality occurred in up to 20% of patients (Eder et al, 1986). In subsequent studies in patients without prior chemotherapy for metastases, approximately 50% achieved remission and, provocatively, a minority of these remained durable (Peters et al, 1988; Ghallie et al, 1994).

Investigators next turned to a strategy based on an interpretation of the work of Norton and Simon (1986). According to their kinetic model, cancer cells grew and regressed – not in the strictly exponential fashion that had previously been proposed (Skipper and Schabel, 1982) but in a Gompertzian manner. The essential feature of Gompertzian kinetics is that the growth fraction of the tumour is not constant, as would be predicted by the exponential model, but rather varies with the size of the tumour. As tumours grow larger, their growth rate decreases. They further hypothesized that the rate of regression of a cancer was related to the dose of chemotherapy administered and to the growth rate of the unperturbed tumour at that phase of its growth curve. Thus, small tumours should be relatively more sensitive to chemotherapy than large ones. Paradoxically, the very high growth rate of very small, subclinical tumours makes their eradication difficult to achieve. Thus, according to Norton and Simon (1986), a phase of 'late intensification' of therapy would be necessary to eradicate a tumour that had been cytoreduced by prior conventional chemotherapy. In the 1970s and 1980s, many groups had studied this approach (using doses substantially less intensive than those achieved with autografting) in patients with different cancers. While some of these trials were positive, the benefit was generally modest (Perloff et al, 1996). The fact that this strategy did not have a greater curative impact might indicate that the level of late intensification achieved in these studies was insufficiently high to have a meaningful impact on drug resistance. Thus, autografting, which facilitated substantial dose-escalation, and the Norton-Simon model seemed to be made for one other. A further theoretical advantage of the 'standard-dose induction' – 'high-dose consolidation' approach was that it identified patients with resistant disease who were known not to benefit substantially from high-dose therapy.

This strategy became the dominant model, and it produced relatively consistent results. Overall and complete response rates were approximately 80–90% and 50–70% respectively. Most of these remissions still ended in relapse, however, and the results were not convincingly superior to those that had been reported for high-dose chemotherapy without prior induction. Nevertheless, the occurrence of durable remissions in 10–20% of patients suggested the possibility that this might be a curative treatment for a minority of patients with metastatic disease (Kennedy et al, 1991; Antman et al, 1992; Crown et al, 1995) There was, of course, a substantial potential for selection bias in these single-arm studies, and there was general acceptance of the need for prospective randomized trials (Henderson, 1990). However, even if these results were to be confirmed, high-dose therapy would remain a poor treatment for metastatic disease, with only a small minority of patients achieving durable remissions. With the decline in treatment-related mortality that occurred following the introduction of peripheral blood progenitors, (Gianni et al, 1989; Brugger et al, 1993; Peters et al, 1993*a*) relapse from complete remission had in fact emerged as the leading cause of failure.

An interpretation of these results based on the Goldie–Coldman model would suggest that these relapses were inevitably due to the persistence, or emergence, of diverse clones of cancer cells with varying drug resistance mechanisms (Goldie and Coldman, 1979). In an attempt to overcome this problem, Gianni et al (1992) devised innovative regimens which sequentially delivered high

*Received 23 July 1996*
*Revised 23 September 1996*
*Accepted 25 September 1996*

*Correspodence to:* J Crown doses of different single agents that were putatively susceptible to different drug-resistance mechanisms (Patrone et al, 1995). Both the 'induction–consolidation' and the 'high-dose sequential models' are, however, based on the premise that populations of cells that are sensitive to a treatment can be efficiently eradicated by a single application of that treatment – a hypothesis which is not entirely consistent with classical chemotherapy theory and practice (Crown and Norton, 1995). The cure of Hodgkin's disease (DeVita et al, 1970) and testis cancer (Einhorn et al, 1977) resulted from the identification of highly active regimens and the application of a sufficient number of cycles of those regimens to eradicate the cancer. As the high-dose programmes used with stem-cell support appear to be the most active regimens that are currently available for the treatment of metastatic breast cancer, would it not be logical to treat patients with multiple cycles of these highly active regimens rather than prefacing their use with treatments that, in the context of cure, are highly ineffective?

This approach might in fact be more consistent with the Norton–Simon model, the ultimate logic of which is that all treatment courses should be given in high dose. Furthermore, the observation that Gompertzian kinetics predicted rapid regrowth of small volume residual tumours argues for abbreviated treatment intervals. In the 1970s (when this hypothesis was first advanced), haematopoietic support technology was rudimentary, and a single cycle of high-dose therapy was as much as most, but not all, (Dunphy and Spitzer, 1992) investigators attempted. Thus induction – consolidation was the most feasible adaptation of the model at that point in time. The introduction of peripheral blood progenitors subsequently facilitated the investigation of multi-cycle high-dose therapy at either standard or accelerated treatment intervals. This strategy is now under investigation (Crown et al, 1992, 1993, 1994; Ayash et al, 1994; Fennelly et al, 1995, Vahdat et al, 1995; Rodenhuis et al, 1996). It is against this historical and theoretical backdrop that we should consider the results of the first two randomized studies of high-dose chemotherapy in metastatic breast cancer.

Bezwoda et al (1995) randomly assigned patients with chemotherapy-naive metastatic disease to receive either conventionally-dosed mitoxantrone, vincristine and cyclophosphamide or high-doses of cyclophosphamide, etoposide and mitoxantrone without induction therapy. The high-dose treatment produced significantly superior response and survival. The study was relatively small, and a disproportionate number of patients on the high-dose arm received tamoxifen post chemotherapy. In addition, patients on the low-dose arm had rather poor survival. It is, however, interesting to note that many of the patients on the high-dose arm were not hospitalized for complications of cytopenia, a finding which suggests that this high-dose regimen was less intensive that those that had been used in other studies.

The second study (Peters et al, 1996) was a test of the classic induction – high-dose consolidation model. Patients with metastatic breast cancer who had achieved a complete response to conventional chemotherapy were randomized to undergo high-dose therapy as immediate consolidation or to receive no further treatment until they relapsed; 'salvage' high-dose chemotherapy was then applied. The cohort who underwent consolidation had a highly significantly prolonged disease-free survival compared with those who were transplanted at relapse, supporting the concept of 'late-intensification'. Paradoxically, the latter group of patients had superior overall survival. While this apparently confusing observation requires explanation and confirmation, high-dose chemotherapy was, again, associated with 5-year survival in a proportion of patients, including non-randomized patients who were transplanted in partial response. A further implication of these results is that a reconsideration of this therapy in relapsed metastatic disease may be required.

It may be that these two trials taken collectively show us that high-dose therapy is indeed more active than low-dose therapy, but that the induction – consolidation model might not represent its most efficient use. While these studies partly establish the credibility of high-dose therapy, conventionally dosed chemotherapy has also improved in recent years, and it is essential that the conventional arms of future confirmatory trials should also be optimized. In one such initiative, the European Breast Cancer Dose-Intensity Study (EBDIS), patients receive docetaxel and anthracycline followed by either cyclophosphamide methotrexate 5-fluorouracil (CMF) or two autograft-supported high-dose cycles. In future studies, investigators will likely address the relative merits of the different high-use strategies and the potential impact of graft engineering (Shpall et al, 1994; Brugger et al, 1995). The impact of high-dose chemotherapy may also be greater in the setting of high-risk stage II–III disease in which the tumour burden is much smaller than it is in patients with clinically overt metastases. Promising results have been reported from single-arm studies (Gianni et al, 1992; Peters et al, 1993*b*), and this approach is now the subject of large international randomized trials.

The last year has been an exciting one for students of high-dose therapy. We may be justified in believing that the first randomized trials have brought us to the 'end of the beginning'. Hopefully, current studies may herald the 'beginning of the end'.

### REFERENCES

Antman K, Ayash LJ, Elias A, Wheeler C, Hunt M, Eder JP, Teicher BA, Critchlow J, Bibbo J, Schnipper LE and Frei E (1992) A phase II study of high dose cyclophosphamide, thiotepa and carboplatin with autologous marrow support in patients with measurable advanced breast cancer responding to standard-dose therapy. *J Clin Oncol* **10**: 102–110

Ayash L, Elias A, Wheeler C, Reich E, Schwartz G, Mazanet R, Tepler I, Warren D, Lynch C, Gonin R, Schnipper L, Frei E and Antman K (1994) Double dose-intensive chemotherapy with autologous marrow and peripheral-blood progenitor-cell support for metastatic breast cancer: a feasibility study. *J Clin Oncol* **12**: 37

Bastholt L, Dalmark M, Gjedde S, Pfeiffer P, Pedersen D, Sandberg E, Kjaer M, Mouridsen HT, Rose C, Nielsen O, Jakobsen P and Bentzen S (1996) Dose-response relationship of epirubicin in the treatment of postmenopausal patients with metastatic breast cancer: a randomized study of epirubicin at four different dose levels performed by the Danish Breast Cancer Cooperative Group. *J Clin Oncol* **14**: 1146–1155

Bezwoda WR, Seymour L and Dansey RD (1995) High-dose chemotherapy with hematopoietic rescue as primary treatment for metastatic breast cancer: a randomized trial. *J Clin Oncol* **13**: 2483–2489

Brugger W, Birken R, Bertz H, Hecht T, Pressler K, Frisch J, Schulz G, Mertelsman R and Kanz L (1993) Peripheral blood progenitor cells mobilized by chemotherapy plus granulocyte-colony stimulating factor accelerate both neutrophil and platelet recovery after high-dose VP16, ifosfamide and cisplatin. *Br J Haematol* **84**: 402–407

Brugger W, Heimfeld S, Berenson RJ, Mertelsmann R and Kanz L (1995) Reconstitution of hematopoiesis after high-dose chemotherapy by autologous progenitor cells generated ex vivo. *N Engl J Med* **333**: 283–287

Cold S, Jensen NV, Brincker H and Rose C (1993) The influence of chemotherapy on survival after recurrence in breast cancer – a population-based study of patients treated in the 1950s, 1960s and the 1970s. *Eur J Cancer* **29A**: 1146–1152

Crown J and Norton L (1995) Potential strategies for improving the results of high-dose chemotherapy in patients with metastatic breast cancer. *Ann Oncol* **6**(suppl. 4): s21–s26

© Cancer Research Campaign 1997

Crown J, Wasserheit C, Hakes T, Fennelly D, Reich L, Moore M, Schneider J, Curtin J, Rubin SC, Reichman B, Moore M, Yao TJ, Gilewski T, Gulati S, Markman M and Norton L (1992) Rapid delivery of multiple high-dose chemotherapy courses with G-CSF and peripheral blood-derived haemopoietic progenitor cells. *J Natl Cancer Inst* **84**: 1935–1936

Crown J, Raptis G, Vahdat L, Fennelly D, Hamilton N and Norton (1993) Sequential high-dose cyclophosphamide, L-PAM and thiotepa in patients with metastatic breast cancer. *Ann Oncol* **5** (suppl. 8): 32

Crown J, Kritz A, Vahdat L, Reich L, Moore M, Hamilton N, Schneider J, Harrison M, Gilewski T, Hudis C, Gulati S and Norton L (1994) Rapid administration of multiple cycles of high-dose myelosuppressive chemotherapy in patients with metastatic breast cancer. *J Clin Oncol* **11**: 1144–1149

Crown JP, Hamilton N, Raptis G, Kritz A, Vahdat L and Norton L (1995) Carboplatin and etoposide in metastatic breast cancer. *Ann Oncol* **6**: 403–405

Decker DA, Ahmann DL and Bisel HF (1979) Complete responders to chemotherapy in metastatic breast cancer. *JAMA* **242**: 2075–2079

DeVita VT, Serpick AA and Carbone PP (1970) Combination chemotherapy in the treatment of advanced Hodgkin's Disease. *Ann Intern Med* **73**: 881–895

Dunphy F and Spitzer G (1992) Use of very-high-dose chemotherapy with autologous bone marrow transplantation in treatment of breast cancer. *J Natl Cancer Inst* **84**: 128–129

Eder JP, Antman K, Peters WP, Henner WD, Elias A, Shea T, Schryber S, Andersen J, Come S, Schnipper L and Frei e (1986) High-dose combination alkylating agent chemotherapy with autologous marrow support for metastatic breast cancer. *J Clin Oncol* **4**: 1592–1597

Einhorn LH and Donohue J (1977) Cis-diamminedichloroplatinum, vinblastine and bleomycin combination chemotherapy in disseminated testicular cancer. *Ann Intern Med* **87**: 293–298

Fennelly D, Schneider J, Spriggs D, Bengala C, Hakes T, Reich L, Barakat R, Curtin J, Moore MAS, Hoskins W, Norton L and Crown J (1995) Dose-escalation of taxol with high-dose cyclophosphamide, with analysis of progenitor cell mobilization and hematologic support of advanced ovarian cancer patients receiving rapidly sequenced high-dose carboplatin/cyclophoisphamide courses. *J Clin Oncol* **13**: 1160–1166

Forastiere AA, Hakes TB, Wittes JT et al (1982) Cisplatin in the treatment of metastatic breast carcinoma. A prospective randomized trial of two dosage schedules. *Am J Clin Oncol* **5**: 243–247

Ghallie R, Richman CM, Adler SA, Cobleigh MA, Korenblit AD, Manson SD, McLeod BC, Taylor SG, Valentino LA, Wolter J and Kaizer H (1994) Treatment of metastatic breast cancer with a split-course high-dose chemotherapy regimen and autologous bone marrow transplantation. *J Clin Oncol* **12**: 342–346

Gianni A, Siena S, Bregni M, Tarella C, Stern A, Pileri A and Bonadonna G (1989) Granulocyte-macrophage colony-stimulating factor to harvest circulating haemopoietic stem cells for autotransplantation. *Lancet* **11**: 580–585

Gianni AM, Siena S and Bregni M (1992) Growth factor supported high-dose sequential adjuvant chemotherapy in breast cancer with >10 positive nodes. *Proc Am Soc Clin Oncol* **11**: 60

Goldie J and Coldman AJ (1979) A mathematical model for relating the drug sensitivity of tumors to their spontaneous mutation rate. *Cancer Treat Rep* **63**: 1727–1773

Henderson IC (1991) Window of opportunity. *J Natl Cancer Inst* **83**: 894–896

Hortobagyi GN, Bodey GP, Buzdar AU, Frye D, Legha SS, Malik R, Smith TL, Blumenschein GR, Yap H-Y and Rodriguez V (1987) Evaluation of high-dose versus standard FAC chemotherapy for advanced breast cancer in protected environment units: a prospective randomized trial. *J Clin Oncol* **5**: 354–364

Kennedy MJ, Beveridge RA, Rowley SD, Gordon GB, Abeloff MD, Davidson NE (1991) High-dose chemotherapy with reinfusion of purged autologous bone marrow following dose-intense induction as initial therapy for metastatic breast cancer. *J Natl Cancer Inst* **83**: 920–926

Norton L and Simon R (1986) The Norton–Simon hypothesis revisited. *Cancer Treat Rep* **70**: 63–169

Patrone F, Ballestrero A, Ferranda F, Brema F, Maraglio L, Valbonesi M, Basta P, Ghio R, Gobbi M and Sessarego M (1995) Four-step high-dose sequential chemotherapy with double hematopoietic progenitor-cell rescue for metastatic breast cancer. *J Clin Oncol* **13**: 840–846

Perloff M, Norton L, Korzun A, Wood W, Carey R, Gottlieb A, Aust J, Bamk A, Silver R, Saleh F, Canellos G, Perry M, Weiss R and Holland J (1996) Postsurgical adjuvant chemotherapy of stage II breast carcinoma with or without crossover to a non-cross-resistant regimen: a cancer and leukaemia group B study. *J Clin Oncol* **14**: 1589–1598

Peters WP, Shpall EJ, Jones RB, Olsen GA, Bast RC, Gockerman JP and Moore JO (1988) High dose combination alkylating agents with bone marrow support as initial treatment for metastatic breast cancer. *J Clin Oncol* **6**: 1368–1376

Peters WP, Rosner G, Ross M, Vredenburgh J, Meisenberg B, Gilbert C and Kurtzberg J (1993*a*) Comparative effects of granulocyte-macrophage colony-stimulating factor (GM-CSF) and granulocyte colony-stimulating factor (G-CSF) on priming peripheral blood progenitor cells for use with autologous bone marrow after high-dose chemotherapy. *Blood* **81**: 1709–1719

Peters WP, Ross M and Vredenburgh JJ (1993*b*) High-dose chemotherapy and autologous bone marrow support as consolidation after standard-dose adjuvant therapy for high risk primary breast cancer. *J Clin Oncol* **11**: 1132–1144

Peters WP, Jones RB, Vredenburgh J, Shpall EJ, Hussein A, Elkordy M, Rubin P, Ross M and Berry D (1996) A large prospective randomized trial of high-dose alkylating agents (CPB) with autologous cellular support (ABMS) as consolidation for patients with metastatic breast cancer achieving complete remission after intensive doxorubicin-based induction therapy (AFM) (abstract 149). *Proc Am Soc Clin Oncol* **15**: 121

Rodenhuis S, Westermann A, Holtkamp MJ, Nooijen WJ, Baars JW, van der Wall E, Slaper-Cortenbach ICM and Schornagel JH (1996) Feasibility of multiple courses of high-dose cyclophosphamide, thiotepa and carboplatin for breast cancer or germ cell cancer. *J Clin Oncol* **14**: 1473–1483

Shpall EJ, Jones RB, Bearman SI, Franklin WA, Archer PG, Curiel T, Bitter M, Claman HN, Stemmer S, Purdy M, Myers SE, Hami L, Taffs S, Heimfeld S, Hallagan J and Berenson R (1994) Transplantation of enriched CD34-positive autologous marrow into breast cancer patients following high-dose chemotherapy: influence of CD34-positive peripheral-blood progenitors and growth factors on engraftment. *J Clin Oncol* **12**: 28–36

Skipper HE and Schabel FM (1982) Quantitative and cytokinetic studies in experimental tumor systems in Holland. *In Cancer Medicine*, Frei J (ed.), pp. 663–684. Lea and Febiger: Philadelphia.

Teicher BA, Holden SA and Cucchi CA Combination thiotepa and cyclophosphamide in vivo and in vitro. *Cancer Res* **48**: 94–100

Vahdat L, Raptis G, Fennelly D, Hamilton N, Reich L, Tiersten A, Harrison M, Hudis C, Moore M, Yao TJ, Norton L and Crown J (1995) Rapidly cycled courses of high-dose alkylating agents supported by filgrastim and peripheral blood progenitor cells in patients with metastatic breast cancer. *Clin Cancer Res* **1**: 1267–1273

© Cancer Research Campaign 1997


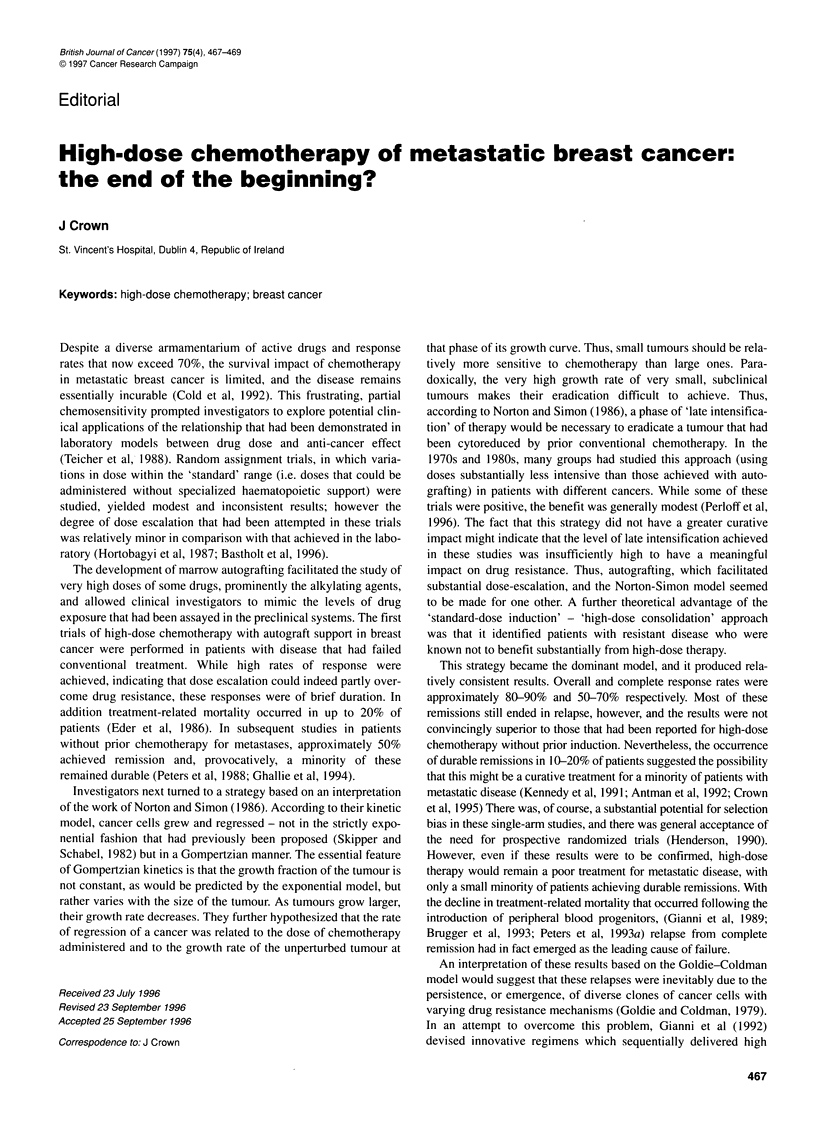

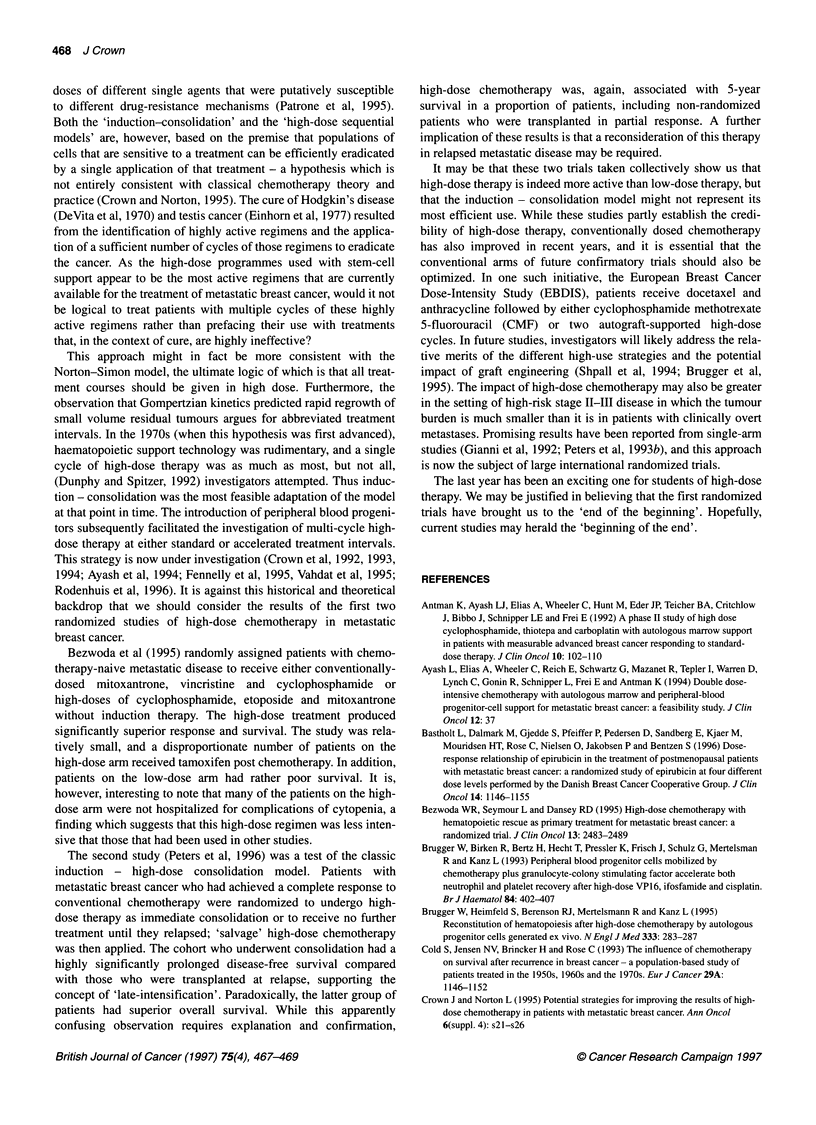

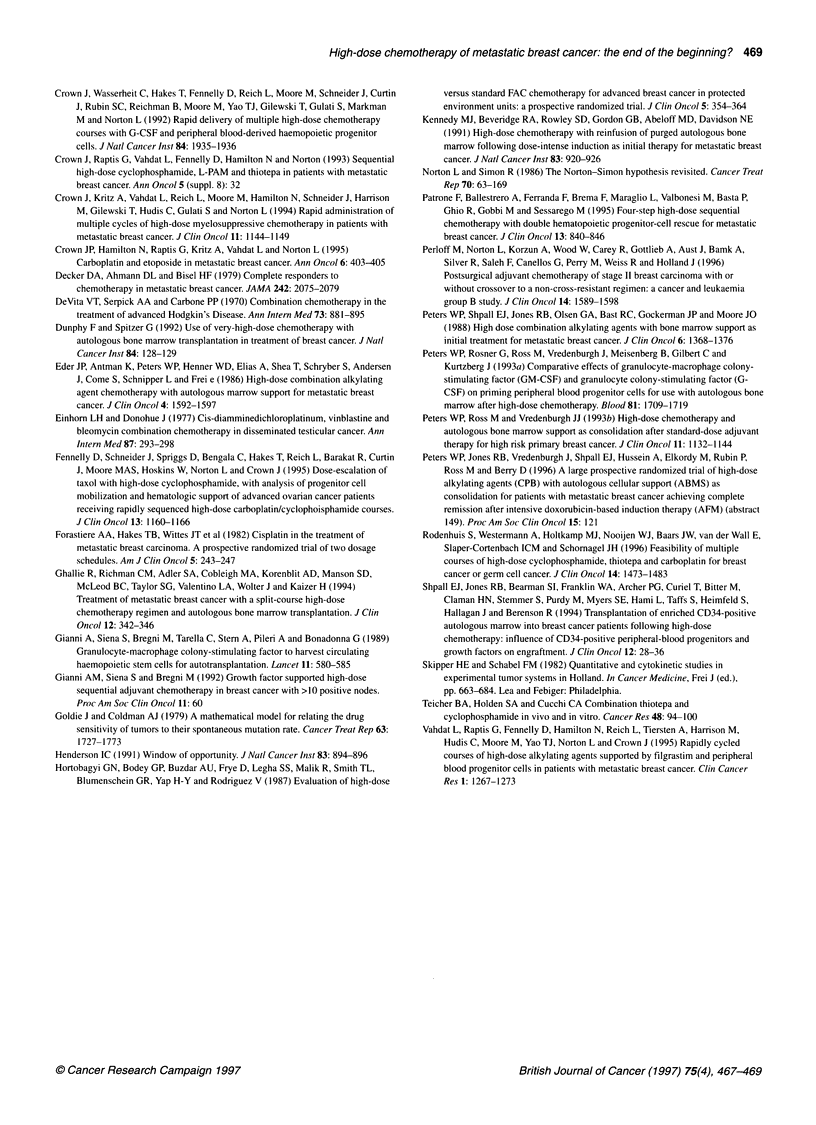


## References

[OCR_00216] Antman K., Ayash L., Elias A., Wheeler C., Hunt M., Eder J. P., Teicher B. A., Critchlow J., Bibbo J., Schnipper L. E. (1992). A phase II study of high-dose cyclophosphamide, thiotepa, and carboplatin with autologous marrow support in women with measurable advanced breast cancer responding to standard-dose therapy.. J Clin Oncol.

[OCR_00224] Ayash L. J., Elias A., Wheeler C., Reich E., Schwartz G., Mazanet R., Tepler I., Warren D., Lynch C., Gonin R. (1994). Double dose-intensive chemotherapy with autologous marrow and peripheral-blood progenitor-cell support for metastatic breast cancer: a feasibility study.. J Clin Oncol.

[OCR_00232] Bastholt L., Dalmark M., Gjedde S. B., Pfeiffer P., Pedersen D., Sandberg E., Kjaer M., Mouridsen H. T., Rose C., Nielsen O. S. (1996). Dose-response relationship of epirubicin in the treatment of postmenopausal patients with metastatic breast cancer: a randomized study of epirubicin at four different dose levels performed by the Danish Breast Cancer Cooperative Group.. J Clin Oncol.

[OCR_00242] Bezwoda W. R., Seymour L., Dansey R. D. (1995). High-dose chemotherapy with hematopoietic rescue as primary treatment for metastatic breast cancer: a randomized trial.. J Clin Oncol.

[OCR_00247] Brugger W., Birken R., Bertz H., Hecht T., Pressler K., Frisch J., Schulz G., Mertelsmann R., Kanz L. (1993). Peripheral blood progenitor cells mobilized by chemotherapy plus granulocyte-colony stimulating factor accelerate both neutrophil and platelet recovery after high-dose VP16, ifosfamide and cisplatin.. Br J Haematol.

[OCR_00256] Brugger W., Heimfeld S., Berenson R. J., Mertelsmann R., Kanz L. (1995). Reconstitution of hematopoiesis after high-dose chemotherapy by autologous progenitor cells generated ex vivo.. N Engl J Med.

[OCR_00267] Castella J., Buj J., Puzo C., Antón P. A., Burgués C. (1995). Diagnosis and staging of bronchogenic carcinoma by transtracheal and transbronchial needle aspiration.. Ann Oncol.

[OCR_00261] Cold S., Jensen N. V., Brincker H., Rose C. (1993). The influence of chemotherapy on survival after recurrence in breast cancer--a population-based study of patients treated in the 1950s, 1960s and 1970s.. Eur J Cancer.

[OCR_00294] Crown J., Hamilton N., Raptis G., Kritz A., Vahdat L., Norton L. (1995). Carboplatin and etoposide in metastatic breast cancer.. Ann Oncol.

[OCR_00288] Crown J., Kritz A., Vahdat L., Reich L., Moore M., Hamilton N., Schneider J., Harrison M., Gilewski T., Hudis C. (1993). Rapid administration of multiple cycles of high-dose myelosuppressive chemotherapy in patients with metastatic breast cancer.. J Clin Oncol.

[OCR_00276] Crown J., Wassherheit C., Hakes T., Fennelly D., Reich L., Moore M., Schneider J., Curtin J., Rubin S. C., Reichman B. (1992). Rapid delivery of multiple high-dose chemotherapy courses with granulocyte colony-stimulating factor and peripheral blood-derived hematopoietic progenitor cells.. J Natl Cancer Inst.

[OCR_00297] Decker D. A., Ahmann D. L., Bisel H. F., Edmonson J. H., Hahn R. G., O'Fallon J. R. (1979). Complete responders to chemotherapy in metastatic breast cancer. Characterization and analysis.. JAMA.

[OCR_00301] Devita V. T., Serpick A. A., Carbone P. P. (1970). Combination chemotherapy in the treatment of advanced Hodgkin's disease.. Ann Intern Med.

[OCR_00304] Dunphy F. R., Spitzer G. (1992). Use of very-high-dose chemotherapy with autologous bone marrow transplantation in treatment of breast cancer.. J Natl Cancer Inst.

[OCR_00309] Eder J. P., Antman K., Peters W., Henner W. D., Elias A., Shea T., Schryber S., Andersen J., Come S., Schnipper L. (1986). High-dose combination alkylating agent chemotherapy with autologous bone marrow support for metastatic breast cancer.. J Clin Oncol.

[OCR_00315] Einhorn L. H., Donohue J. (1977). Cis-diamminedichloroplatinum, vinblastine, and bleomycin combination chemotherapy in disseminated testicular cancer.. Ann Intern Med.

[OCR_00320] Fennelly D., Schneider J., Spriggs D., Bengala C., Hakes T., Reich L., Barakat R., Curtin J., Moore M. A., Hoskins W. (1995). Dose escalation of paclitaxel with high-dose cyclophosphamide, with analysis of progenitor-cell mobilization and hematologic support of advanced ovarian cancer patients receiving rapidly sequenced high-dose carboplatin/cyclophosphamide courses.. J Clin Oncol.

[OCR_00330] Forastiere A. A., Hakes T. B., Wittes J. T., Wittes R. E. (1982). Cisplatin in the treatment of metastatic breast carcinoma: A prospective randomized trial of two dosage schedules.. Am J Clin Oncol.

[OCR_00335] Ghalie R., Richman C. M., Adler S. S., Cobleigh M. A., Korenblit A. D., Manson S. D., McLeod B. C., Taylor S. G., Valentino L. A., Wolter J. (1994). Treatment of metastatic breast cancer with a split-course high-dose chemotherapy regimen and autologous bone marrow transplantation.. J Clin Oncol.

[OCR_00343] Gianni A. M., Siena S., Bregni M., Tarella C., Stern A. C., Pileri A., Bonadonna G. (1989). Granulocyte-macrophage colony-stimulating factor to harvest circulating haemopoietic stem cells for autotransplantation.. Lancet.

[OCR_00353] Goldie J. H., Coldman A. J. (1979). A mathematic model for relating the drug sensitivity of tumors to their spontaneous mutation rate.. Cancer Treat Rep.

[OCR_00358] Henderson I. C. (1991). Window of opportunity.. J Natl Cancer Inst.

[OCR_00360] Hortobagyi G. N., Bodey G. P., Buzdar A. U., Frye D., Legha S. S., Malik R., Smith T. L., Blumenschein G. R., Yap H. Y., Rodriguez V. (1987). Evaluation of high-dose versus standard FAC chemotherapy for advanced breast cancer in protected environment units: a prospective randomized study.. J Clin Oncol.

[OCR_00367] Kennedy M. J., Beveridge R. A., Rowley S. D., Gordon G. B., Abeloff M. D., Davidson N. E. (1991). High-dose chemotherapy with reinfusion of purged autologous bone marrow following dose-intense induction as initial therapy for metastatic breast cancer.. J Natl Cancer Inst.

[OCR_00374] Norton L., Simon R. (1986). The Norton-Simon hypothesis revisited.. Cancer Treat Rep.

[OCR_00378] Patrone F., Ballestrero A., Ferrando F., Brema F., Moraglio L., Valbonesi M., Basta P., Ghio R., Gobbi M., Sessarego M. (1995). Four-step high-dose sequential chemotherapy with double hematopoietic progenitor-cell rescue for metastatic breast cancer.. J Clin Oncol.

[OCR_00385] Perloff M., Norton L., Korzun A. H., Wood W. C., Carey R. W., Gottlieb A., Aust J. C., Bank A., Silver R. T., Saleh F. (1996). Postsurgical adjuvant chemotherapy of stage II breast carcinoma with or without crossover to a non-cross-resistant regimen: a Cancer and Leukemia Group B study.. J Clin Oncol.

[OCR_00398] Peters W. P., Rosner G., Ross M., Vredenburgh J., Meisenberg B., Gilbert C., Kurtzberg J. (1993). Comparative effects of granulocyte-macrophage colony-stimulating factor (GM-CSF) and granulocyte colony-stimulating factor (G-CSF) on priming peripheral blood progenitor cells for use with autologous bone marrow after high-dose chemotherapy.. Blood.

[OCR_00406] Peters W. P., Ross M., Vredenburgh J. J., Meisenberg B., Marks L. B., Winer E., Kurtzberg J., Bast R. C., Jones R., Shpall E. (1993). High-dose chemotherapy and autologous bone marrow support as consolidation after standard-dose adjuvant therapy for high-risk primary breast cancer.. J Clin Oncol.

[OCR_00394] Peters W. P., Shpall E. J., Jones R. B., Olsen G. A., Bast R. C., Gockerman J. P., Moore J. O. (1988). High-dose combination alkylating agents with bone marrow support as initial treatment for metastatic breast cancer.. J Clin Oncol.

[OCR_00421] Rodenhuis S., Westermann A., Holtkamp M. J., Nooijen W. J., Baars J. W., van der Wall E., Slaper-Cortenbach I. C., Schornagel J. H. (1996). Feasibility of multiple courses of high-dose cyclophosphamide, thiotepa, and carboplatin for breast cancer or germ cell cancer.. J Clin Oncol.

[OCR_00428] Shpall E. J., Jones R. B., Bearman S. I., Franklin W. A., Archer P. G., Curiel T., Bitter M., Claman H. N., Stemmer S. M., Purdy M. (1994). Transplantation of enriched CD34-positive autologous marrow into breast cancer patients following high-dose chemotherapy: influence of CD34-positive peripheral-blood progenitors and growth factors on engraftment.. J Clin Oncol.

[OCR_00447] Vahdat L., Raptis G., Fennelly D., Hamilton N., Reich L., Tiersten A., Harrison M., Hudis C., Moore M., Yao T. J. (1995). Rapidly cycled courses of high-dose alkylating agents supported by filgrastim and peripheral blood progenitor cells in patients with metastatic breast cancer.. Clin Cancer Res.

